# Pathogen-epithelium interactions and inflammatory responses in *Salmonella* Dublin infections using ileal monolayer models derived from adult bovine organoids

**DOI:** 10.1038/s41598-024-62407-2

**Published:** 2024-05-20

**Authors:** Minae Kawasaki, Craig S. McConnel, Claire R. Burbick, Yoko M. Ambrosini

**Affiliations:** 1 Department of Veterinary Clinical Sciences, College of Veterinary Medicine, Washington State University, Pullman, WA USA; 2 Department of Veterinary Microbiology and Pathology, College of Veterinary Medicine, Washington State University, Pullman, WA USA

**Keywords:** Adult stem cells, Bovine ileum, In vitro infection model, Organoid-derived monolayer, *Salmonella*, *S.* Dublin, Bacterial host response, Gastrointestinal models

## Abstract

*Salmonella enterica* serovar Dublin (*S.* Dublin) is an important enteric pathogen affecting cattle and poses increasing public health risks. Understanding the pathophysiology and host–pathogen interactions of *S.* Dublin infection are critical for developing effective control strategies, yet studies are hindered by the lack of physiologically relevant in vitro models. This study aimed to generate a robust ileal monolayer derived from adult bovine organoids, validate its feasibility as an in vitro infection model with *S.* Dublin, and evaluate the epithelial response to infection. A stable, confluent monolayer with a functional epithelial barrier was established under optimized culture conditions. The model’s applicability for studying *S.* Dublin infection was confirmed by documenting intracellular bacterial invasion and replication, impacts on epithelial integrity, and a specific inflammatory response, providing insights into the pathogen-epithelium interactions. The study underscores the utility of organoid-derived monolayers in advancing our understanding of enteric infections in livestock and highlights implications for therapeutic strategy development and preventive measures, with potential applications extending to both veterinary and human medicine. The established bovine ileal monolayer offers a novel and physiologically relevant in vitro platform for investigating enteric pathogen-host interactions, particularly for pathogens like *S.* Dublin.

## Introduction

*Salmonella enterica* subspecies e*nterica* serovar Dublin (*S.* Dublin) is a cattle-adapted serovar that infect not only bovine but other animals including humans. It is a globally important enteric pathogen with increasing public health risks^1^. Outcome of *S.* Dublin infection in cattle range from subclinical, leading to latent carrier state, to clinical, causing systemic illness such as enteritis and sepsis^2,3^. In humans, *S.* Dublin infection usually carries more devastating health impact, causing severe bloodstream infections such as septicemia, osteomyelitis, meningitis, and even death in susceptible populations^1,4^. A recent study has documented increasing incidence of *S.* Dublin infection and resulting disease severity in humans as well as emerging antimicrobial drug resistance among *S.* Dublin isolates^1^.

Typically, human infections with *S.* Dublin are transmitted through direct contact with infected animals or consumption of contaminated beef and dairy products^1,5^. It has been postulated that development of antimicrobial resistance in *S.* Dublin could be associated with the use of such drugs in cattle^1^. Therefore, development of effective disease control strategies in cattle, hence better understanding of the pathophysiology and host–pathogen interactions, are critical in limiting economic and public health impacts associated with *S.* Dublin infection. Nonetheless, such studies have been hampered by the lack of physiologically relevant in vitro infection models which allow effective evaluations of host–pathogen interactions at the intestinal luminal interface.

Historically, *Salmonella* infection of intestinal epithelial cells have been investigated extensively using in vivo murine and immortalized cell line-based in vitro models^6^. In cattle, in vivo models have been utilized to explore host–pathogen interactions and host response to *Salmonella* infection^7,8^. More recently, with the emergence of organoid technology, in vitro organoid-based infection models have been developed for cattle using various pathogens including *Salmonella*^9–11^. Three-dimensional (3D) organoid models are considered superior to traditional cell line-based models due to their structural, cellular and functional resemblance to in vivo tissue^10^. However, accessibility to the apical surface of the epithelial cells where host–pathogen interactions take place is limited due to their luminal structure, posing a significant challenge to such studies^6,12,13^.

To overcome this limitation, organoid-derived 2D monolayer system, and various infection models thereof, have been developed and utilized to investigate host response to and interactions with pathogens mainly in humans and mice^14–16^. Adoption of the 2D monolayer system using cells derived from 3D organoids provides accessible luminal interface, while retaining other key features of the 3D organoid model such as cellular heterogeneity and polarization. While such models offer unique opportunities to better understand pathophysiology of enteric infections, studies on bovine organoid-derived monolayers remain limited^11,17,18^. Recognizing that most existing studies, including the one by Sutton, et al., focus primarily on calves^10,11,17^, the present study targets adult bovine models. This approach is crucial as adult cattle are the main source of beef and dairy products and are likely to have different physiological responses to infection and treatment compared to younger animals, thereby providing insights more relevant to the conditions under which human exposure occurs.

The development and validation of the adult bovine ileal monolayer model of *S.* Dublin infection represent a significant advancement with far-reaching implications for public health. By facilitating a deeper understanding of the pathophysiology of *S.* Dublin infections and the host–pathogen interactions at the intestinal epithelium, this model directly contributes to the development of more effective strategies for disease control and prevention in cattle crucial for mitigating the risk of zoonotic transmission to humans. Given that many human infections with *S.* Dublin are linked to direct contact with infected animals or consumption of contaminated animal products, improving disease management in cattle can significantly reduce the incidence of such infections in humans, thereby protecting public health.

Therefore, the present study aimed to (1) describe a robust technique to generate ileal monolayers derived from adult bovine organoids, (2) confirm feasibility of the ileal organoid-derived monolayers as an in vitro infection model with *S.* Dublin, and (3) evaluate the impact of *S.* Dublin infection on the epithelial cells and their response to *S.* Dublin infection. The use of organoid-derived monolayers in studying species specific host-microbial interactions underscores the importance of adopting a One Health approach. Collaborative efforts across disciplines to achieve the best health outcomes for people, animals, and the environment recognize the health of each is interconnected and dependent on the others. Therefore, the contributions of this study extend beyond the immediate field of veterinary science, offering valuable insights and tools to protect and improve human health as part of a comprehensive public health strategy.

## Results

### Generation and characterization of stable ileal monolayers derived from adult bovine organoids

A stable intestinal epithelial monolayer was generated using adult bovine ileal organoids, as previously described^19^. The optimized culture conditions for the ileal monolayer were determined by varying the culture medium compositions and cell seeding densities (Supplementary Fig. S1). Confluent monolayers were consistently formed within 2–3 days of culture with a seeding density of 5 × 10^5^ cells/well in a 24-well cell culture insert and in an optimized monolayer culture medium consisting of organoid culture medium supplemented with Rho-associated kinase (ROCK) and transforming growth factor beta (TGFB) inhibitors and 20% fetal bovine serum (FBS). A stable transepithelial electrical resistance (TEER) was reached and consistently maintained for up to 9 days with the optimized protocol. In contrast, the time required to form a confluent monolayer was more variable from 2 to 6 days and a stable TEER was maintained only for 2 days when the cells were seeded at a lower density (3 × 10^5^ cells/well). Similarly, formation of a confluent monolayer was delayed or lacked stability when the cells were cultured in the other test media including a commercially available organoid culture medium (IntestiCult) and DMEM/F12-based organoid culture medium with or without supplementation and less than 20% FBS. Therefore, bovine ileal organoid-derived monolayers were generated by seeding the cells at a density of 5 × 10^5^ cells/well and culturing in the optimized monolayer culture medium consisting of organoid culture medium supplemented with 20% FBS and including ROCK and TGFB inhibitors in the subsequent study.

**Figure 1:**
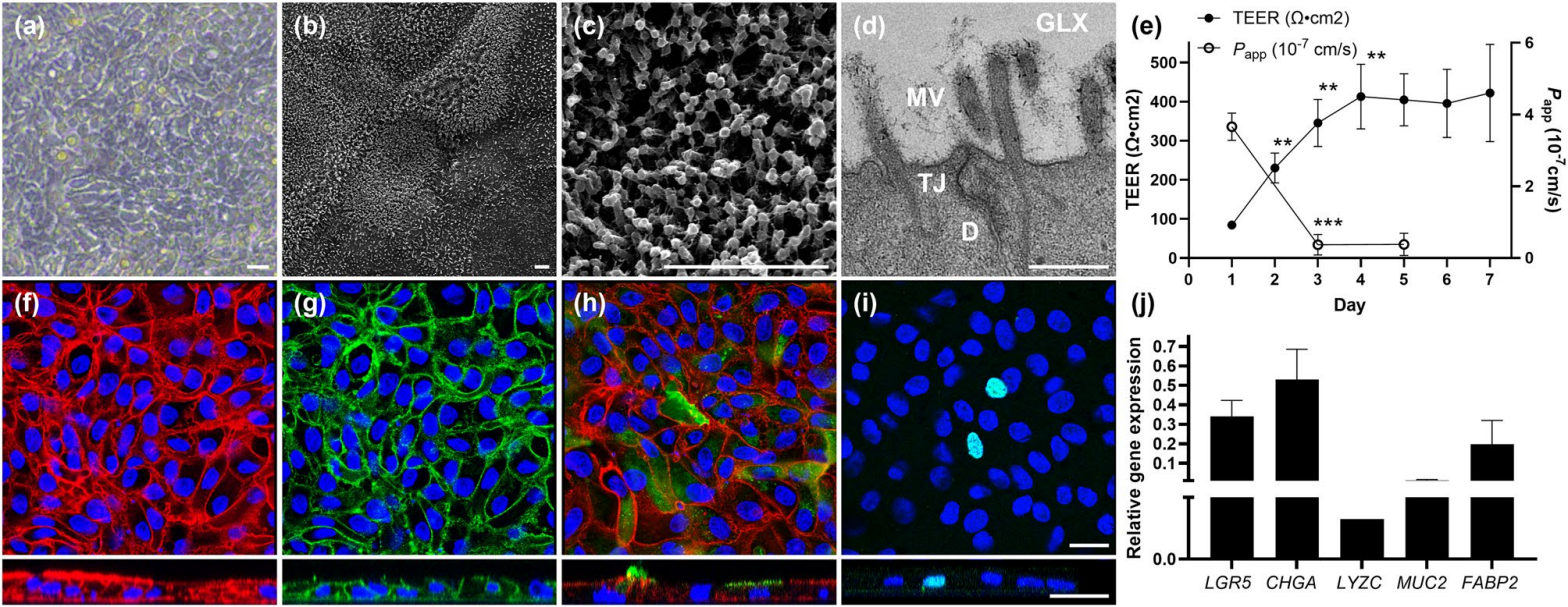
Characterization of adult bovine ileal organoid-derived monolayers. (**a**) A representative phase-contrast image of the monolayer at Day 6 of culture, exhibiting characteristic cobblestone morphology. Bar: 20 μm. (**b**,**c**) Representative scanning electron microscopy images of the monolayers at Day 6 of culture in low (**b**) and high (**c**) magnifications. The entire surface of the monolayer was uniformly covered by densely packed microvilli. Bars: 2 μm. (**d**) A representative transmission electron microscopy image of the monolayer at Day 5 of culture, demonstrating the formation of apical microvilli (MV) covered with glycocalyx (GLX), inter-cellular tight junctions (TJ) and desmosome (D). Bar: 500 nm. (**e**) The transepithelial electrical resistance (TEER) measured daily from Day 1 to 7 and apparent permeability (*P*_app_) of fluorescein isothiocyanate-dextran across the monolayer measured at Day 1, 3, and 5 of culture. Results are expressed as mean ± s.e.m. obtained from three independent experiments with at least two technical replicates per experiment using three biological replicates. **p < 0.01, ***p < 0.001 compared with the previous day. (**f**–**i**) Immunofluorescence staining of the monolayers against F-actin (f, red), E-cadherin (g, green), *Sambucus nigra* agglutinin (SNA) (h, green), and EdU (i, cyan) at Day 6 of culture. Nuclei were stained with DAPI (blue). Top-down view (top) and cross-sectional images (z-stack, bottom) are shown. Bars: 20 μm. (**j**) RT-qPCR of the cells within the monolayers at Day 4 of culture. Relative expression levels of stem and lineage cell marker genes were evaluated using *GAPDH*, *RPL0* and *ACTB* as internal controls. Results are expressed as mean ± s.e.m. obtained from two technical replicates from three biological replicates. *LGR5* leucine rich repeat containing G protein-coupled receptor 5, *CHGA* chromogranin A, *LYZC* lysozyme C, *MUC2* mucin 2, *FABP2* fatty acid-binding protein 2, *GAPDH*: glyceraldehyde-3-phosphate dehydrogenase, *RPL0* ribosomal protein L0 and *ACTB* β-actin.

The monolayers cultured under the optimized conditions exhibited uniform cobblestone morphology on phase-contrast microscopy (Fig. 1a). Scanning electron microscopy (SEM) revealed densely packed microvilli uniformly covering the entire surface of the monolayer (Fig. 1b,c). Transmission electron microscopy (TEM) demonstrated the presence of apical microvilli with associated glycocalyx and the formation of epithelial cell junction structures, namely tight junctions and desmosome (Fig. 1d). All of these structures are characteristics of intestinal epithelial cells and in accordance with previous observations^20,21^.

Daily evaluation of TEER across the monolayer documented significant increase relative to the previous day until Day 4 of culture (*p* < 0.01), reaching the value of 412.6 ± 82.7 Ω*cm^2^ from 84.4 ± 7.9 Ω*cm^2^ measured at Day 1. Subsequently, a plateau of approximately 400 Ω*cm^2^ was maintained for more than 7 days (Fig. 1e). Paracellular permeability assay using 4 kDa fluorescein isothiocyanate (FITC)-dextran also documented initial significant decline in the apparent permeability (*P*_app_) from Day 1 to 3 of culture (*p* < 0.001), which was maintained at Day 5 (Fig. 1e). These observations indicated the establishment and maintenance of functional and stable epithelial barrier integrity throughout the culture period.

Furthermore, immunofluorescence staining of confluent monolayers against F-actin and E-cadherin demonstrated uniform expressions of these proteins at cell-to-cell junctions (Fig. 1f,g). Z-stack images of these markers revealed strong apical expression of F-actin and basolateral expression of E-cadherin together with basally located DAPI-stained nuclei. These findings are consistent with cellular structures noted in a polarized epithelium characterized by a F-actin-rich apical brush border, E-cadherin-containing basolateral adherens junctions and basal nuclei^17,22^. Staining against *Sambucus nigra* agglutinin (SNA), a sialic acid-specific lectin which binds to mucin produced by goblet cells, and EdU confirmed presence of functioning goblet cells and proliferative cells within the monolayers, respectively (Fig. 1h,i). Cells within the monolayers also exhibited expression of stem and epithelial lineage cell marker genes confirmed by RT-qPCR analysis (Fig. 1j). Taken together, these findings support the presence of multilineage cell populations within the bovine ileal organoid-derived monolayers.

### Establishment of bovine ileal organoid-derived monolayers as an in vitro infection model

**Figure 2:**
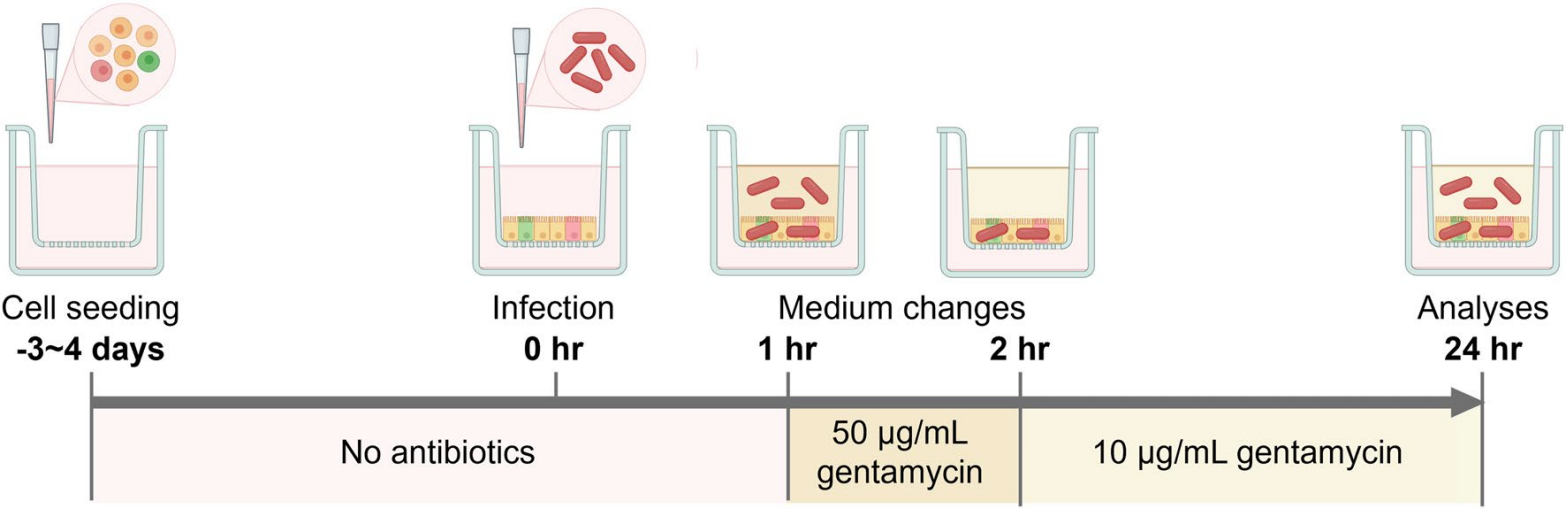
A schematic of experimental design for the infection of bovine ileal organoid-derived monolayers with *S*. Dublin. An experimental timeline from generation of ileal monolayers to infection with *S.* Dublin and subsequent analyses is shown. Single cells derived from adult bovine ileal organoids are seeded onto 24-well cell culture inserts utilizing the optimized monolayer culture media without antibiotics. Once the formation of confluent monolayers is confirmed by stable TEER measurements (3–4 days after seeding), the epithelial cells are exposed apically with 1 × 10^6^ CFU/well of *S.* Dubin for 1 h in antibiotic-free culture medium, facilitating bacterial invasion of the epithelial cells. The control cells are treated with the same volume of the monolayer culture medium without bacteria. After the invasion period, the monolayers undergo a treatment phase to eliminate non-invaded bacteria. Initially, this involves an hour-long incubation with a medium containing 50 μg/mL of gentamicin, aimed at killing extracellular bacteria. Following this, the monolayers are maintained in a medium supplemented with 10 μg/mL of gentamicin for the remainder of the experiment (24 h post infection). This step ensures the suppression of any residual bacteria, preventing their overgrowth in the culture medium. Created with BioRender.com.

Confluent, stable ileal organoid-derived monolayers were inoculated with *S.* Dublin into the apical compartment. After initial optimization (Supplementary Fig. S2), monolayers were cocultured with *S.* Dublin at an infection concentration of 1 × 10^6^ CFU/well for 1 h in an antibiotic-free monolayer culture medium to allow bacterial adhesion and invasion into epithelial cells prior to treatment with 50 and 10 μg/mL gentamicin at 1- and 2 h post infection to eliminate extracellular bacteria and facilitate subsequent examination of internalized bacterial survival and replication, respectively (Fig. 2). Feasibility of adult ileal organoid-derived monolayers as an in vitro infection model was confirmed by documenting interactions between *S.* Dublin and the ileal epithelial cells with multimodal analyses.

**Figure 3:**
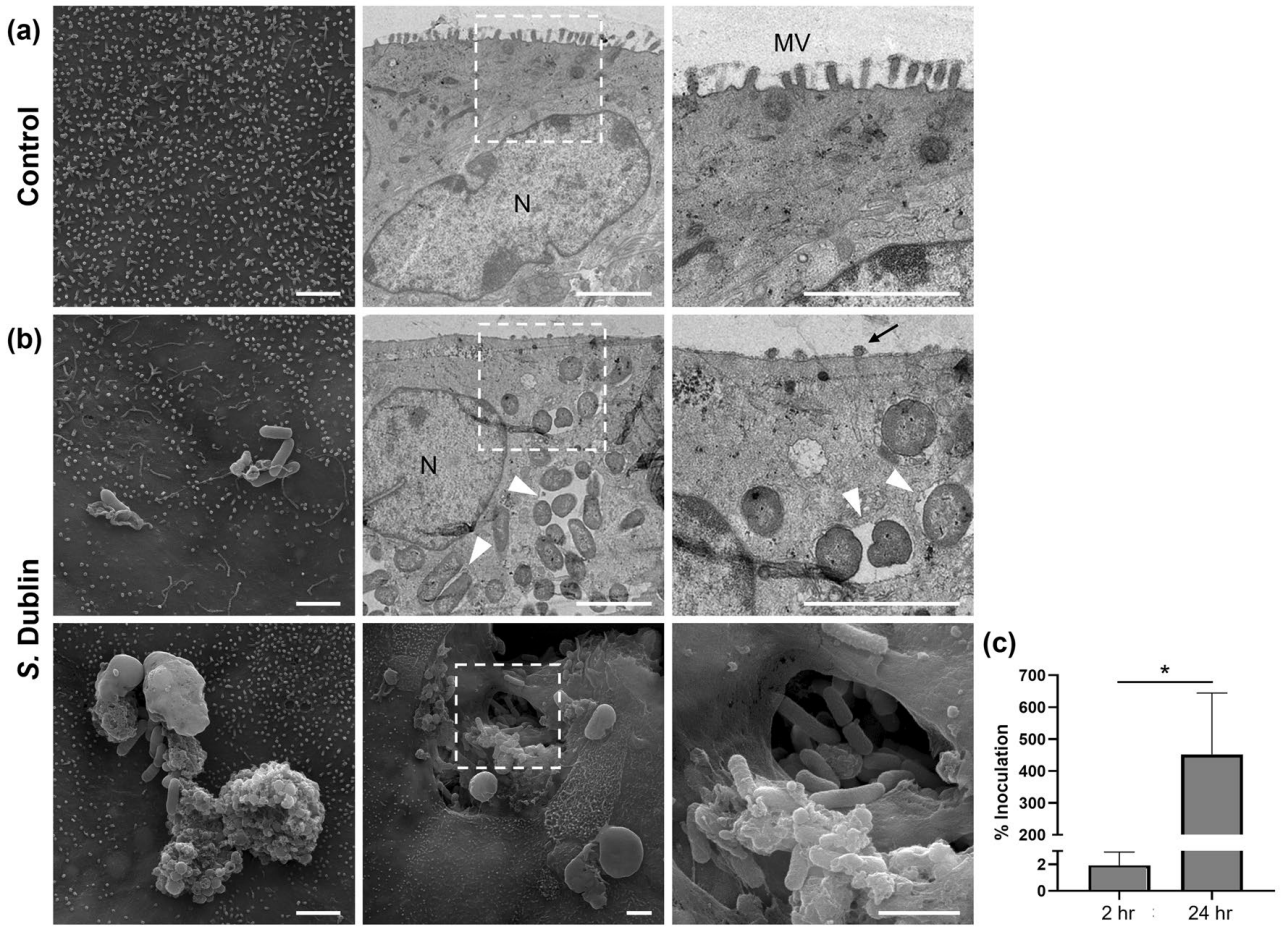
Invasion and replication of *S.* Dublin in bovine ileal organoid-derived monolayers. (**a**) Representative scanning (SEM, left) and transmission (TEM, center and right) electron microscopy images of uninfected monolayers. Apical surface of the monolayer is covered with uniform microvilli. (**b**) Representative SEM (top left and bottom) and TEM (top center and right) images of *S.* Dublin-infected monolayers at 24 h post infection. SEM revealed adhering bacteria on the apical surface of the monolayer (top left) and mucus (bottom left) as well as invading bacteria into the epithelial cell through disruption of the apical membrane (bottom center and right). TEM revealed intracellular bacteria within vesicles in the cytoplasm of the epithelial cell (arrowheads) and effacement of microvilli on the apical surface of the monolayer (arrow). The right column images show magnification of the white dashed area in both (**a**) and (**b**). N: nuclei, MV: microvilli. Bars: 2 μm. (**c**) Enumeration of intracellular bacteria at 2- and 24 h post infection relative to the inoculum. Results are expressed as mean ± s.e.m. obtained from three independent experiments with two technical replicates per experiment using three biological replicates. *p < 0.05.

SEM imaging at 24 h post infection revealed bacterial adhesion and microvillous effacement on the apical surface of the epithelial cells in the *S.* Dublin-infected monolayers compared with uniform microvilli noted in the control monolayers (Fig. 3a,b). TEM imaging revealed intracellular bacteria encapsulated within vesicles, or endosomes, in the cytoplasm of the infected monolayers. Furthermore, the interactions between the epithelial cells and *S.* Dublin were observed as both bacteria adhering to mucus and disruption of the apical membrane in association with intracellular invasion. Pseudo-coloring of the SEM image effectively highlighted *S*. Dublin invading, replicating within and being released from epithelial cells through disruption of the host cell structure (Supplementary Fig. S3). These observations were consistent with those reported previously using traditional in vivo and in vitro models^8,15,23–25^.

Enumeration of intracellular bacteria at 2- and 24 h post infection revealed the efficiency of intracellular invasion during the first hour of exposure to the monolayer was 1.9 ± 0.9%, and intracellular survival over the 24 h study period was 451.8 ± 175.8% of the inoculum, respectively (*p* < 0.05) (Fig. 3c). The total number of bacteria within cells and those released into the apical compartment increased by more than 900-fold over the 24 h period relative to 2 h post infection. Overnight culture of the cell culture medium collected from the apical and basolateral compartments at 2 h post infection grew no identifiable bacterial colonies (Supplementary Table S1). These results confirmed that *S.* Dublin can invade, replicate and survive in the adult bovine ileal epithelial cells, indicating that the current monolayer system recreated the environment allowing *S.* Dublin to establish intracellular infection.

### Impact of *S*. *Dublin* infection on epithelial barrier integrity

**Figure 4:**
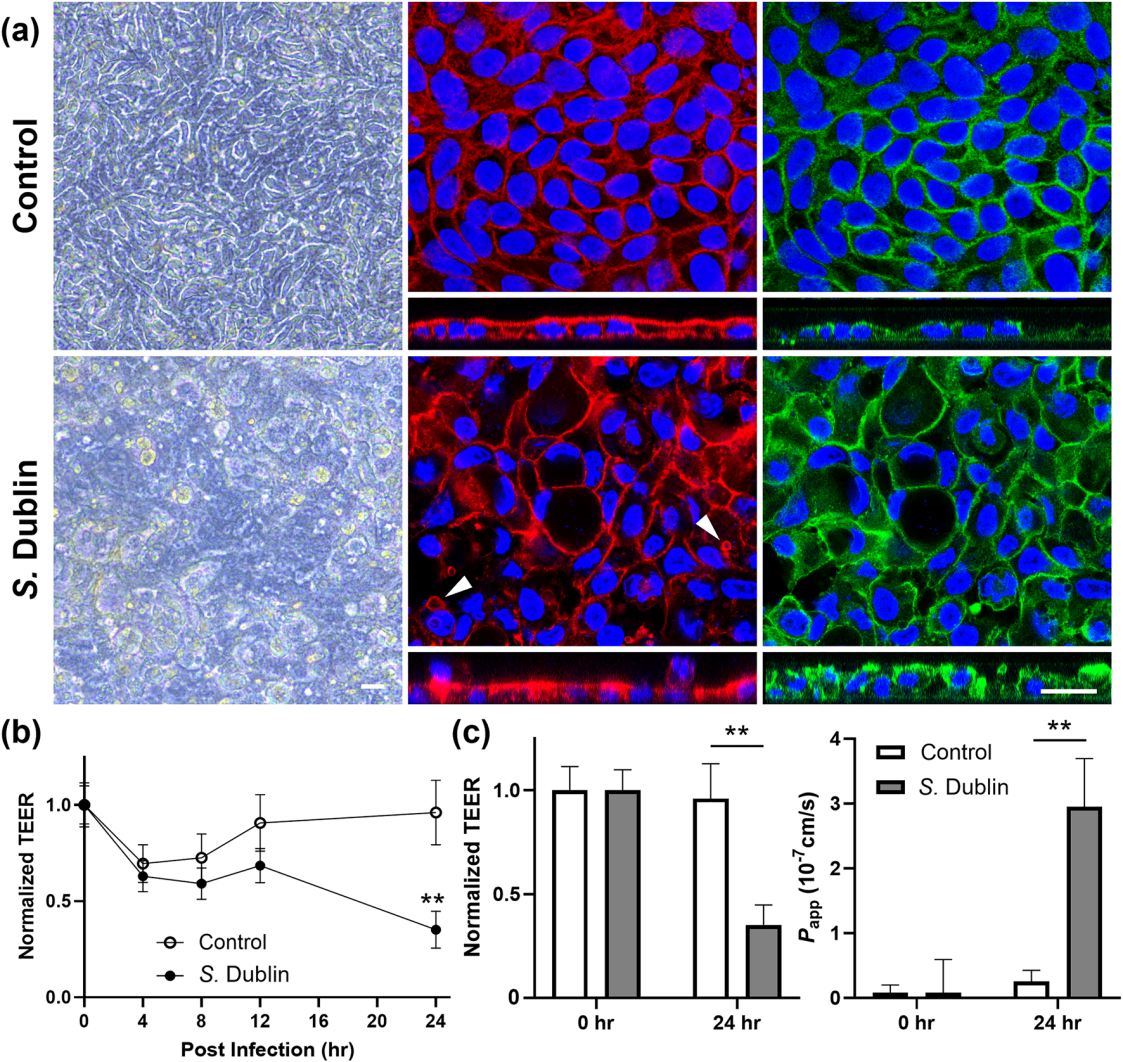
Impacts of *S.* Dublin infection on epithelial barrier integrity of the bovine ileal organoid-derived monolayers. (**a**) Representative phase-contrast (left) and immunofluorescent images (center: F-actin, red and right: E-cadherin, green) of uninfected (top) and infected (bottom) monolayers at 24 h post infection. Nuclei were stained with DAPI (blue). Top-down view and cross-sectional images are shown. Arrowheads indicate contractile actin rings. Bars: 20 μm. (**b**) Changes in the TEER relative to the pre-infection value over the course of the experiment. TEER was measured at 0-, 4-, 8-, 12- and 24 h post infection with *S.* Dublin, normalized by the pre-infection value, and compared between the control and infected monolayers at each time point. (**c**) Normalized TEER (left) and *P*_app_ (right) values at 0- and 24 h post infection. Significant differences were noted between control and *S*. Dublin-infected monolayers at 24 h in both TEER and *P*_app_. For both (**b**) and (**c**), results are expressed as mean ± s.e.m. obtained from four independent experiments with at least two technical replicates per experiment using three biological replicates. **p < 0.01.

Phase-contrast microscopy at 24 h post infection with *S.* Dublin revealed extensive epithelial damage characterized by disruption of the uniform monolayer accompanied by cellular extrusion, while the uninfected control maintained an undisrupted monolayer with uniform cobblestone morphology (Fig. 4a). Immunofluorescence staining revealed disruption of F-actin and formation of contractile actin rings in the infected monolayers similar to previous observations using human colorectal cell lines^23,26^. Extrusion of epithelial cells from the apical surface of the monolayers was also evident in z-stack image of the infected monolayer. Additionally, *S.* Dublin infection caused distinct accumulation and redistribution of adherens junction protein E-cadherin, contrasting to the uniform distribution observed in the controls as reported previously^24,27^. Pleomorphism of epithelial cells, observed as greater variation in cellular shape, was also evident in the *S.* Dublin infected monolayers, comparing with more regular cell morphology noted in the uninfected controls.

The measurement of TEER before and after *S.* Dublin infection documented significant decline in infected monolayers (normalized TEER: 0.35 ± 0.10) compared with the uninfected controls (0.96 ± 0.17) at 24 h post infection (*p* < 0.01) (Fig. 4b,c). Significantly compromised barrier integrity in *S*. Dublin-infected monolayers aligns with the phase-contrast microscopy observation. This change led to a marked increase in *P*_app_ in the *S*. Dublin-infected monolayers compared with the control (2.95 ± 0.42 × 10^–7^ vs 0.26 ± 0.74 × 10^–7^ cm/s, *p* < 0.01) at 24 h post infection (Fig. 4c).

### Immune response of ileal epithelial cells to *S*. *Dublin* infection

**Figure 5:**
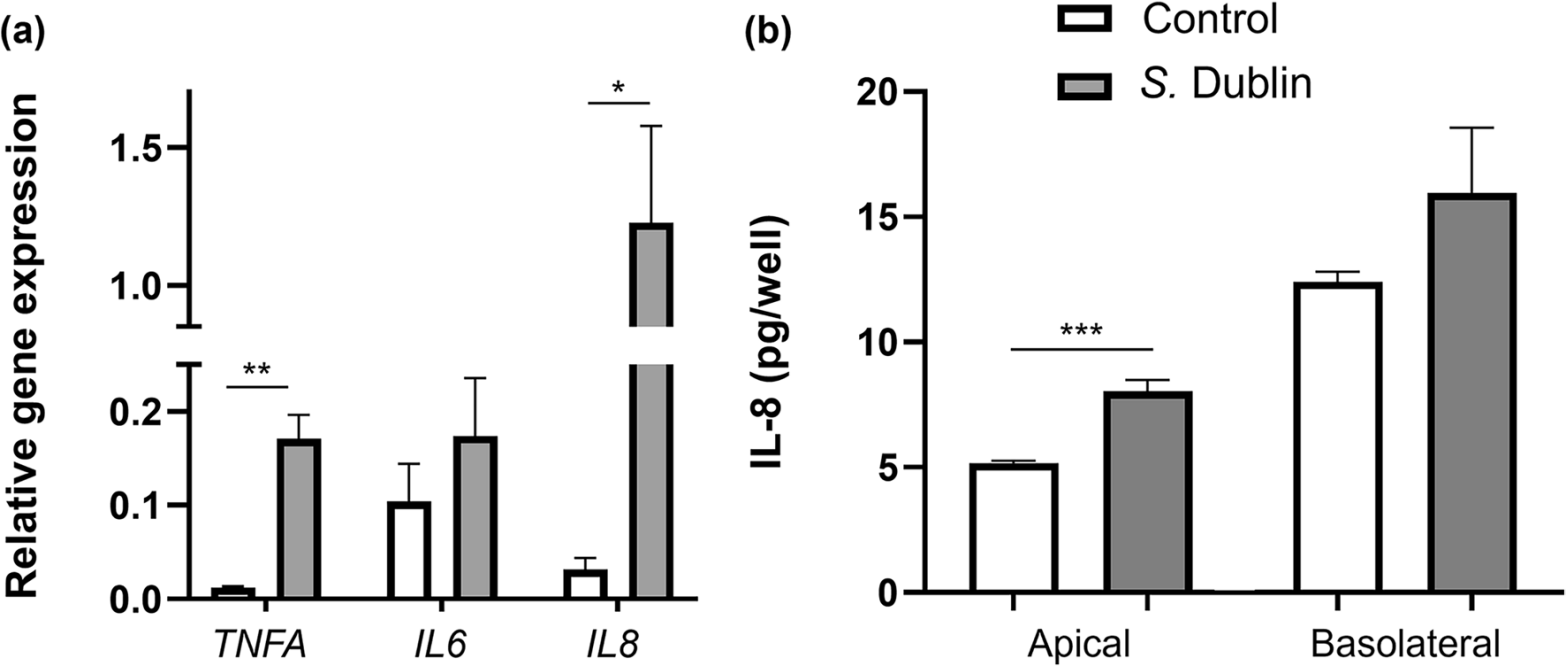
Immune response of bovine ileal organoid-derived monolayers to *S.* Dublin infection. (**a**) RT-qPCR of the cells within the monolayers at 24-h post infection. Relative gene expressions for cytokines (*TNFA*, *IL6*, and *IL8*) were evaluated using *GAPDH*, *RPL0* and *ACTB* as internal controls. (**b**) IL8 concentrations in the apical and basolateral culture media were determined with ELISA. For both evaluations, results are expressed as mean ± s.e.m obtained from three independent experiments with two technical replicates per experiment using three biological replicates and compared between the control and infected monolayers. *TNFA* tumor necrosis factor alpha, *IL6* interleukin 6, *IL8* interleukin 8. *p < 0.05, **p < 0.01, ***p < 0.001.

Immune response of the ileal monolayers to *S.* Dublin infection was evaluated using RT-qPCR and ELISA against inflammatory cytokines. RT-qPCR revealed significant upregulation of *TNFA* (*p* < 0.01) and *IL8* (*p* < 0.05), but not *IL6* (*p* = 0.22), genes in the infected monolayers compared to the uninfected controls (Fig. 5a). Levels of TNFA released into the culture media fell below the assay range (< 0.123 ng/mL) in both apical and basolateral compartments using a commercial bovine TNFA ELISA kit. Levels of IL8 was higher in both apical and basolateral culture media collected from the *S.* Dublin-infected wells relative to those detected in the control wells, with significant difference being noted in the apical medium (*p* < 0.001) (Fig. 5b). Approximately 2/3 of the total IL8 secreted from the cells were detected in the basolateral culture media in both the control and infected wells, indicating preferential secretion of IL8 toward the basolateral direction as described previously^28,29^. The ELISA assay for IL6 was not carried out due to the absence of a significant increase in RT-qPCR results.

## Discussion

The present study described the establishment of a stable adult bovine ileal organoid-derived monolayer and confirmed feasibility of the system as a valuable in vitro model to study *S.* Dublin infection and host response to the bacteria. Successful use of DMEM/F12-based culture media in both organoids and monolayer cultures improved cost-efficiency of the technique, hence accessibility to the technology, compared with commercially available organoid culture medium as described previously^11^. Multimodal imaging analyses utilizing immunofluorescence staining of F-actin, E-cadherin, SNA and EdU, combined with SEM and TEM revealed apical brush border with microvilli and glycocalyx and epithelial cell junction structures at lateral inter-cellular borders validating the cellular architecture and functional orientation expected in in vivo intestinal epithelial cells.

The organoid-derived monolayer system is superior to the conventional cell-based monolayer models because it retains cellular heterogeneity derived from intestinal stem cells similar to the native intestinal tissue. Relatively abundant expression of the enteroendocrine cell marker gene and lower expressions of Paneth cell and goblet cell marker genes are in line with previous observations with bovine ileal organoids^19,30^. Additionally, the organoid-derived monolayer system offers significant advantages for in vitro studies of host–pathogen interactions compared to 3D organoid models. Its 2D structure provides direct and unrestricted access to the apical surface of epithelial cells, facilitating more effective infection studies, which resolves the challenges posed by the complex and enclosed structure of 3D organoids^9,11^. Lastly, monolayers ensure more consistent seeding densities and reduce variability in the host–pathogen ratio, enhancing both experimental reproducibility and the reliability of quantitative assessments, such as TEER measurements. These features make monolayers particularly valuable for examining the impacts of enteric pathogens on host epithelial cells.

The infection model described here successfully recapitulated multiple observations reported in in vivo and in vitro studies investigating host–pathogen interactions and host response to *Salmonella* infection in the intestine. These observations include intracellular invasion, survival and replication of bacteria, effacement and perturbation of microvilli on the apical surface of the epithelial cells, disruption of an intact monolayer accompanied by extrusion of infected epithelial cells, rearrangement of inter-cellular tight junctions, decline in the TEER value, and increased inflammatory response^15,23–25,31–34^. Robustness of the technique, as confirmed by employing multimodal imaging and analytical techniques, not only offers a valuable tool for investigation of bovine intestinal epithelial cell biology but also contributes to broadening the currently limited knowledge of bovine organoids and associated technologies.

The present study successfully established and maintained a stable monolayer most consistently and for longer culture duration when the cells were seeded at a higher density (5 × 10^5^ cells/well). This observation was different from the previous studies which reported higher success rate with much lower seeding density (2.5 × 10^4^ cells/well vs. 1 ~ 2.5 × 10^5^ cells/well) using calf and porcine ileal organoids^17,35^. Contrary to these studies, lower cell numbers in our experiments led to patchy, island-like growth without achieving full confluency (data not shown). Additionally, this study used 20% FBS media supplementation, also used in porcine ileal and rabbit duodenal monolayers^35–37^, whilst 1% FBS has been reported effective with calf ileal monolayers^17^. Other species, including humans, dogs, and mice, typically do not require the addition of FBS to culture organoids and organoid-derived monolayers^14,38,39^. This variability in FBS requirements between species underscores the need to optimize culture conditions based on specific biological characteristics of the tissue origin, further complicating direct comparisons across different studies. It is possible that age of donors at the time of tissue sampling might influence cell survival and formation of confluent monolayers. Furthermore, stability of the adult monolayers, which was reached earlier and maintained longer with slightly higher TEER value in the present culture condition compared with those reported in calves, suggests adult cells may mature faster and be more stable than calf cells. These features could provide additional advantage to some studies where a wider window for subsequent experiments is ideal.

Paracellular permeability assay was applied for the first time to bovine intestinal monolayers and validated the functionality of the epithelial barriers at Day 3 of culture when the TEER value reached ~ 350 Ω*cm^2^. Although the TEER value is a useful indicator of monolayer formation and cellular maturation, a wide variation of the reported values makes it more difficult to ascertain the formation of a functional epithelial barrier^40^. The paracellular permeability assay complements the TEER value by directly evaluating the translocation of tracer molecules across the monolayer. Moreover, comparing TEER values reported from different laboratories does not always provide meaningful information as they are influenced by multiple factors such as temperature and culture conditions including cell types, passage numbers and culture medium compositions^40^. In fact, the stable TEER values observed in this study were much lower than those reported with human and porcine intestinal cell lines and porcine ileal organoid-derived monolayers (> ~ 1000 Ω*cm^2^)^35,41,42^, although they were equivocal to or slightly higher than organoid-derived monolayers in humans and mice (~ 400 Ω*cm^2^)^43,44^ or bovines (~ 300 Ω*cm^2^)^17,18^. The results highlight the importance of coupling different analytical techniques to more accurately interpret observed variations between studies.

The results demonstrated successful *Salmonella* invasion by treating monolayers with high-concentration gentamicin following a 1-hour incubation in an antibiotic-free culture medium to eliminate extracellular bacteria, confirming invasion occurred during the initial phase. Continued treatment with a low concentration of gentamicin inhibited further replication of extruded bacteria without killing them, utilizing its concentration-dependent properties. Enhanced SEM and TEM images, including zoomed-in and pseudo-coloring, visually corroborated the bacterial invasion and replication dynamics within the monolayers, showcasing the effective establishment of *Salmonella* infection model in adult ileal organoid-derived monolayers.

The percentage of *S.* Dublin invading epithelial cells during the first hour of infection was approximately 2% of the inoculum. This result was slightly greater than the previous observations (0.7–1.5%) using human ileal organoid-derived monolayer and cell line-based culture models infected with various strains of *S.* Typhimurium or *S.* Typhi^8,15,45^. Intriguingly, despite the apparent greater efficiency of *S.* Dublin to invade the adult bovine ileal epithelial cells, significant disruption of the epithelial barrier integrity was only detected at 24 h post infection, which was much slower than what has been reported in human colonic cell lines^34^. The less striking and gradual impact of *S.* Dublin infection in the present model could be associated with a potentially superior local immune response generated by the bovine ileal organoid-derived monolayers. For instance, increased inflammatory cytokine release and mucus secretion protecting epithelial cells from bacterial invasion have been suggested by previous studies which documented lower levels of infection in multi-cellular models relative to single cell culture models^8,45^. It has also been proposed that expression of genes or cell surface receptors which influence bacterial invasion or host immune response could have been altered in these models^8,45^. These potential explanations are also applicable to the organoid-derived monolayer systems due to their physiological resemblance to in vivo tissue attributable to their multi-cellular nature as demonstrated by the present and several other studies^11,17,46^.

Bacterial factors, such as host-species and segmental tropism, could also have contributed to the variations among different studies as noted in studies utilizing different segments of human- and chicken-derived intestinal organoids or *Salmonella* pathovars^15,45,47^. Although *S.* Dublin is a cattle-adapted serotype and could cause lesions in the ileum, cecum and colon of infected animals, it can also establish life-long infection in cattle leading to an asymptomatic carrier status especially in healthy mature individuals^3^. Comparison of *S.* Dublin infection in adult- to calf-derived ileal monolayers may or may not result in similar observations to the present adult-derived monolayer model given these differences. Nonetheless, greater than 900-fold increase in the number of bacteria recovered at 24 h relative to that recovered at 2 h post infection is far greater than the previous observation using chicken intestinal organoid models^47^. This result suggests the present ileal monolayer system recreates an in vivo environment allowing bacterial survival and efficient replication, and thus is an effective in vitro infection model to study host–pathogen interactions and host response to *S.* Dublin infection.

Redistribution of adherens junction protein and rearrangement of cytoskeleton actin filament as well as a significant decline in the TEER following exposure to *S.* Dublin were all in line with various in vivo and in vitro studies^23,24,26,27,34,48^. The initial TEER variations observed in both control and *S*. Dublin-infected monolayers were primarily due to physiological adjustments to media changes which occurred at 1- and 2 h post infection. Despite a transient decrease in TEER in the control group, these values stabilized close to the pre-treatment value and remained significantly higher at 24 h compared to the *S*. Dublin-infected group, which showed a marked decrease. This indicates that while medium changes induce some fluctuation, they do not cause a sustained decline in TEER in contrast to the pronounced effect of infection on barrier function. Establishing a correlation between TEER and *P*_app_ values in our system enabled us to infer the *P*_app_ values following *S*. Dublin infection. As expected, a significant decrease in TEER values and a marked increase in *P*_app_ values were observed 24 h after the *Salmonella* infection due to invaded *Salmonella* rupturing the cells and compromising the cell membrane integrity as shown in SEM images (Fig. 3b). Both contraction of actin filaments and redistribution of junctional proteins have been described as dying cell is pushed out of the monolayer while the monolayer tries to maintain its integrity as a part of normal physiological turnover of intestinal epithelial cells^23^. These observations also characterize a process of *Salmonella*-induced barrier dysfunction, which facilitates bacterial translocation across the gut epithelium and subsequent bacterial dissemination^27^. Taken together, these results further confirm that the adult bovine ileal organoid-derived monolayer system is a viable in vitro infection model which can be adopted in various studies to better understand pathophysiology and host–pathogen interactions during challenges with *S.* Dublin and other enteric pathogens in bovine.

*S.* Dublin infection of the adult bovine ileal monolayers resulted in significant upregulation of chemokine *IL8* and proinflammatory cytokines *TNFA* expression as well as secretion of IL8, both apical and basolateral, compared with the uninfected controls. IL8 is a major chemoattractant for neutrophils and plays an important role in the pathogenesis of *Salmonella* infection by inducing an early mucosal inflammatory response at the site of infection. Increased expression and secretion of IL8 has been reported upon invasion of epithelial cells by bacteria including *Salmonella* using various in vivo and in vitro models such as human colonic epithelial cells and bovine ligated ileal loop models^49–51^. The response noted in the present study agreed with these observations and was also consistent with histopathological observations of suppurative enteritis in calves spontaneously infected with *S.* Dublin^52^. This evidence supports the cellular response presented here are likely representing normal physiologic responses of in vivo intestine upon challenge with *S.* Dublin.

Contrasting to *IL8*, upregulation of *TNFA* expression was not associated with increased TNFA secretion. This observation partially agrees with the previous studies, which did not observe significant alterations in expression and secretion of TNFA upon infection with *Salmonella*^49,50^. TNFA is a potent proinflammatory mediator playing a crucial role in host defense against *Salmonella* infection^51^. It is known to be expressed by some epithelial cells and upregulation of *TNFA* has been reported in human intestinal epithelial cells and murine ligated ileal loop models infected with *Salmonella*^28,53^. Explanations for these discrepancies between studies, even when using the models derived from the same species, require further investigation. Moreover, future studies should include the assessment of earlier or later time points to better capture the dynamics of these inflammatory cytokines at both the transcriptional and protein levels. However, these findings underscore the importance of developing in vitro models which exhibit similar response to the species of interest at cellular levels beyond clinical and gross pathological similarities to better understand pathogenesis of *Salmonella*-induced enterocolitis.

Several potential studies and alternative approaches could enhance our current understanding of the interactions between *Salmonella* and host epithelial cells in bovine models. Studies involving delta SPI-1 and/or delta SPI-2 mutant strains of *Salmonella* could provide critical insights into how *Salmonella* invasion affects the integrity of host cell membranes, potentially confirming that only the bacteria that invade host cells can replicate and cause pathogenic cell rupture. Investigating membrane rearrangements during *Salmonella* invasion and the subsequent cell responses could shed light on infection dynamics. Co-staining of bacteria and specific cell types within the monolayers could reveal possible bacterial tropism towards a certain cell type, providing an insight into host-pathogen interactions. Additionally, comparative assessments of microvilli length in ileal tissues and organoid-derived monolayers could enhance understanding of cellular differentiation. Further studies involving use of the growth media described by Sutton et al.^17^ or differentiation-inducing culture medium described by Sato et al.^54^ to explore organoid biology, and incorporating different cell types such as macrophage-like cells and fibroblasts to better simulate in vivo conditions would be valuable. Moreover, analyzing cell toxicity, LDH production, and gene expression through techniques like heatmapping could offer deeper insights into organoid biology and the immune response at both genetic and protein levels, necessitating time-point studies to fully understand these dynamics.

The present study did not evaluate the direct gene expression of the donor tissue; thus, it is not possible to compare and discuss similarities or differences in genetic properties between the monolayers and their originating tissues. Compared with previous research^19^, there are considerable gene expression variations between 2D ileal monolayers and 3D organoids, demonstrating how culture methods influence cellular behavior. Nonetheless, the 2D monolayers are particularly valuable for studying specific aspects of host-pathogen interactions as well as intestinal physiology, providing key insights into these processes.

Having developed a robust and reproducible organoid-derived monolayer model, the study provided valuable tools for screening and developing targeted strategies to prevent and treat bovine infections, thereby advancing veterinary medicine. Additionally, the translational potential into human medicine is significant. Insights from bovine models, particularly concerning intestinal pathogens like *Salmonella*, are applicable to human health due to the major reservoir role that cattle play for *Salmonella* in humans. These cross-species insights have the potential to expedite the development of innovative therapeutic strategies, improving health outcomes for both animals and humans and contributing broadly to public health.

## Conclusion

The present study successfully established a stable, confluent monolayer derived from adult bovine ileal organoids under optimized culture conditions, introducing a novel in vitro platform for investigating intestinal epithelial dynamics and host–pathogen interactions. The high-density cell seeding and supplementation with ROCK and TGFB inhibitors, along with 20% FBS, enabled the formation of a functional epithelial barrier with cell junction structures, demonstrating uniform cobblestone morphology, densely packed microvilli, and robust TEER and *P*_app_ values. The model’s application to *S.* Dublin infection offered insights into bacterial adhesion, invasion, and intracellular replication, leading to significant epithelial disruption and a specific inflammatory response marked by *TNFA* and *IL8* upregulation, thereby highlighting its physiological relevance and suitability for studying enteric pathogens. The findings reveal the potential of organoid-derived monolayers to advance our understanding of infectious diseases in livestock and suggest implications for developing therapeutic strategies and preventive measures. The model also serves as a valuable tool for evaluating drug efficacy and studying host-microbial interactions, relevant to both veterinary and human medicine. Future research should explore the model’s application to other gastrointestinal pathogens, interactions between the intestinal epithelium and the microbiota, immune responses, drug absorption, and metabolism, with comparative studies on monolayers from different intestinal sections and species, to broaden our understanding of intestinal physiology and disease.

## Materials and methods

### Development and maintenance of bovine ileal organoids

Ileal tissue samples were freshly obtained from four approximately 15- to 18 month-old cattle at a local slaughterhouse (Supplementary Table S2). The tissue was sampled and processed for crypt isolation and organoid development following the previously described protocol^19^. Briefly, approximately 4–8 mm sections of ileal tissue were sampled using biopsy forceps and rinsed with ice-cooled Dulbecco’s phosphate-buffered saline (PBS) containing 1 × penicillin/streptomycin and 25 μg/mL gentamicin. The samples were further minced to small fragments and incubated in 20 mM ethylenediaminetetraacetic acid (EDTA) solution at 4 ℃ for 15 min on a tube rotator to enhance crypt release and isolation efficiency. The crypts were collected, resuspended in Matrigel and seeded onto a 48-well plate using 30 μL per well at a density of approximately 30–40 crypts per Matrigel dome. Subsequently, each well received 300 μL of organoid culture medium once Matrigel was polymerized at 37 ℃ for 10–15 min. The organoid culture medium was prepared according to a previous study^19^ (Supplementary Table S3). The medium was changed every other day for growth and maintenance of ileal organoids. Organoids were passaged every 6 to 8 days^19^. Briefly, organoids were recovered from Matrigel using Cell Recovery Solution, dissociated with TrypLE Express, resuspended in Matrigel at an expansion ratio of approximately 1:6 and cultured as described above.

### Generation of ileal organoid-derived monolayers

Ileal organoids which demonstrated stable growth and expansion following at least three passages were used to generate monolayers. Ileal organoids were harvested from Matrigel using Cell Recovery Solution and dissociated with TrypLE Express supplemented with 10 μM Y-27632 at 37 ℃ for 10 min. Dissociated organoids were filtered through a 70 μm cell strainer to obtain single cell suspensions. Subsequently, cells were resuspended in the optimized monolayer culture medium and seeded onto a 24-well cell culture insert with 0.4 μm pore size (Falcon, 0.33 cm^2^) at 5 × 10^5^ cells/well. The cell culture insert was precoated with 2% (v/v) Matrigel in Advanced DMEM/F12 supplemented with 2 mM GlutaMAX and 10 mM HEPES at 37 ℃ for 1 h. The monolayer culture medium was prepared by adding 10 μM Y-27632, 500 nM LY2157299, and 20% (v/v) FBS to the organoid culture medium without antibiotics (Supplementary Table S3). Each well received 200 μL and 500 μL of the monolayer culture medium in apical and basolateral compartments, respectively, and the medium was changed every other day. Formation of confluent monolayers was monitored daily using phase-contrast microscopy (DMi1, Leica). Epithelial barrier integrity of the monolayers was evaluated through TEER measurement and paracellular permeability assay. Structural and cellular characteristics of the monolayers were evaluated using electron microscopy, immunofluorescence staining, and RT-qPCR analyses.

### Bacterial culture and infection of monolayers

*S.* Dublin isolated from a bovine was grown in 3 mL of Luria–Bertani (LB) broth overnight at 37 ℃ with shaking at 200 rpm. The culture was diluted 1:10 into fresh LB broth and cultured for 2 h to late log phase. Bacteria were harvested, washed with PBS, and resuspended to a concentration of 1 × 10^7^ CFU/mL in the monolayer culture medium. The monolayer was infected apically by replacing 100 μL of culture medium with the same volume of bacterial suspension and incubated for 1 h in an antibiotic-free culture medium to allow bacterial invasion to the epithelial cells. Subsequently, the monolayer was incubated with the medium containing 50 μg/mL gentamicin for 1 h to eliminate extracellular bacteria, and then in the medium containing 10 μg/mL gentamicin till the end of the experiment, i.e. 24 h post infection. Similarly, the control monolayer was initially treated with 100 μL of the monolayer culture medium without bacteria and subsequently with the media containing 50 and 10 μg/mL gentamicin for the same duration of time as the *S*. Dublin-infected monolayers.

Infection of the ileal epithelial cells and intracellular multiplication of *S.* Dubin was confirmed by enumerating intracellular bacteria at 2 h post infection following treatment with 50 μg/mL gentamicin, again at 24 h post infection and by electron microscopies at 24 h. Epithelial cell integrity and structure were evaluated by measuring TEER at 0-, 4-, 8-, 12-, and 24 h post infection and by immunofluorescence staining of structural and junctional proteins at 24 h post infection. Response of bovine ileal epithelial cells to *S.* Dublin infection was evaluated by RT-qPCR and ELISA of cytokines at 24 h post infection.

### Bacterial enumeration

Infected monolayers were rinsed with PBS and lysed with 1% Triton X-100 at 2- and 24 hour post infection. The culture media in the apical and basolateral compartments and the PBS used to rinse the apical compartment were also collected at each time point. Serial dilutions of the lysate, apical medium and PBS rinse, and basolateral medium were plated on LB agar in triplicates and incubated at 37 ℃ overnight to determine CFU per well. Percent internalization and intracellular survival of *S.* Dublin were calculated by normalizing the CFU/well obtained from the cell lysate at 2- and 24 h post infection to the initial bacterial inoculum, respectively^45^. The replication rate of *S.* Dublin was calculated by normalizing the total CFU/well obtained from the cell lysate, apical medium and PBS rinse, and basolateral medium at 24 h post infection to that obtained at 2 h post infection. Enumeration was performed in three independent experiments using three biological replicates, with at least two technical replicates per experiment.

### TEER measurement

The TEER of ileal organoid-derived monolayers was monitored daily and at 0- (pre-), 4-, 8-, 12- and 24 h post infection using an Epithelial Voltohmmeter (EVOM) (Millicell ERS-2, Millipore) connected with Ag/AgCl electrodes. The measurement and calculation of TEER followed previously described techniques^40^. Briefly, the resistance across the cell culture insert was measured in wells of both blank (TEER_blank_) and ileal monolayers (TEER_monolayer_) by placing one electrode in the apical compartment and the other in the basolateral compartment without touching the membrane. The measured TEER (Ω) was converted to reported TEER (Ω･cm^2^) by multiplying the difference of these values (TEER_monolayer_—TEER_blank_) by the surface area of the cell culture insert where the cells are cultured (cm^2^). The measurement was obtained in at least three independent experiments using three biological replicates, with at least two technical replicates per experiment to assess reproducibility in various bovine ileal organoid-derived monolayers.

### Paracellular permeability assay

The epithelial barrier integrity of the monolayers was evaluated by performing permeability assay on Days 1, 3 and 5 of culture following the previously described protocol^46,55^. Briefly, 0.5 mg/mL of 4 kDa FITC-dextran was applied to the apical compartment at time 0, and the fluorescence intensity of the culture medium in the basolateral compartment was determined every 20 min over 120 min using SpectraMax i3x microplate reader (molecular devices). The measurements were obtained with excitation and emission wavelengths of 495 and 535 nm, respectively. The *P*_app_ (cm/s) was calculated by dividing the amount of FITC-dextran that passed through the cell layer over a fixed time period (μg/s) by the initial FITC-dextran concentration in the apical compartment (μg/mL) and the surface area of the cell culture insert (cm^2^). The assay was performed in three independent experiments using three biological replicates, with at least two technical replicates per experiment.

The *P*_app_ value following *Salmonella* infection was estimated based on the correlation between TEER and *P*_app_ values in our monolayer system established in the initial experiments. The TEER values measured at different time points over 24 h of infection were normalized by the pre-infection value to demonstrate longitudinal changes. The normalized TEER values at time 0- and 24 h post infection were used to infer the *P*_app_ values.

### Immunofluorescence staining

Monolayers were fixed with 4% paraformaldehyde for 15 min, permeabilized with 0.3% Triton X-100 for 10 min and blocked with 2% bovine serum albumin (BSA) for 60 min at room temperature. Primary antibody and fluorescence probes against E-cadherin (BD Biosciences, 1:200), SNA (Vector Laboratories, 1:100), F-actin (Phalloidin) (Invitrogen, 1:400) and nuclei (DAPI) (Thermo Scientific, 1:1000) were diluted in 2% BSA and incubated for 1 h at room temperature in the dark. Subsequently, the membrane was excised from the inserts and mounted on glass slides using ProLong Gold Antifade reagent. Additionally, EdU staining was performed to detect actively proliferating cells following manufacture’s protocol. Fluorescence images were captured using a white-light point scanning confocal microscope (SP8-X, Leica) with a 63 × objective and images were processed using LAS X software (Leica).

### Electron microscopy

Monolayers were fixed and processed for SEM and TEM as described previously^39,46^. Briefly, monolayers were fixed with 2.5% glutaraldehyde in 0.1 M sodium cacodylate buffer overnight at 4 ℃, rinsed with 0.1 M cacodylate buffer, and post-fixed with 0.5% osmium tetroxide overnight at 4 ℃. Samples were serially dehydrated in 30 to 100% ethanol and finally in hexamethyldisilazane (HMDS) for SEM or in propylene oxide for TEM. SEM samples were coated with Pt/Pd sputter coater (Cressington High Resolution Sputter Coater) and imaged using Quanta 200F SEM (FEI). TEM samples were embedded in Spurrs resin, sectioned to 80 nm thickness, stained with uranyl acetate, potassium permanganate and Raynold’s lead, and imaged using Tecnai G2 20 Twin TEM (FEI).

To highlight the intracellular invasion and replication of bacteria, manual pseudo-coloring of an SEM image was performed using image-editing software (Adobe Inc. Adobe Photoshop). The quick selection tool was used to select bacteria on the original layer. This selection was transferred to a new layer, where the bacteria were highlighted with a contrasting paint color. The layer's opacity was set to 25% to ensure visibility. Edges were refined with the eraser tool to correct any selection inaccuracies.

### RT-qPCR

RT-qPCR was performed as described previously^19^. Briefly, total RNA was extracted from the control and *S.* Dublin-infected monolayers at 24 h post infection using RNeasy Plus Mini Kit (Qiagen) according to the manufacture’s protocol. Removal of the cells from cell culture inserts were facilitated by applying kit cell lysis buffer. High-Capacity cDNA Reverse Transcription Kit (Applied Biosystems) was used to synthesize cDNA. RT-qPCR was performed using PowerUp SYBR Green Master Mix (Applied Biosystems), with the primer sequence being amplified at 60 ℃ for 40 cycles. Expression levels of stem (*LGR5*) and epithelial lineage cell (*CHGA*, *LYZC*, *MUC2*, *FABP2*) marker genes were evaluated using the cDNA synthesized from the uninfected control samples for characterization of the monolayers. Gene expression levels of cytokines (*TNFA*, *IL6*, *IL8*) were compared between the uninfected control and infected monolayers. Primers used in this study were adopted from previous studies and summarized in Supplementary Table S4^10,17,56–58^. Internal controls, namely *GAPDH*, *RPL0*, and *ACTB*^10,58,59^, were used to normalize relative expressions of the target genes, which were calculated by applying the standard curve method. Normalization of the target genes was achieved by taking the mean of three internal control genes for each sample with an aim of enhancing normalization accuracy^60,61^. RT-qPCR reactions were carried out in duplicate from three biological replicates with two technical replicates per experiment.

### ELISA

Culture media of apical and basolateral compartments were collected at 24 h post infection, centrifuged and supernatant was stored at − 80 ℃ until the analysis was carried out. Selection of cytokines for detection using ELISA methods was guided by significant upregulation of gene expression based on RT-qPCR analysis. Commercially available sandwich ELISA kits, namely bovine TNFA ELISA kit (Thermo Scientific) and human IL8 ELISA kit (R&D Systems), were used as reported previously^62,63^. The measurement was obtained with SpectraMax i3x microplate reader (Molecular Devices) according to the manufacture’s protocol. The assays were performed in duplicate from three biological replicates with two technical replicates per experiment.

### Statistical analyses

Quantitative data were analyzed using R v.3.4.1 (The R foundation). The normality of each dataset was evaluated using Shapiro–Wilk test. Subsequently, either paired t-tests or Wilcoxon’s signed rank tests were employed to compare data between the control and *S.* Dublin-infected monolayers. The results were presented as mean ± standard error of the mean (s.e.m.), with *p* < 0.05 being considered statistically significant.

### Supplementary Information

Supplementary Information.

## Data Availability

All data relevant to this study are included in this published article and its Supplementary Information files.

## References

[1] Harvey RR (2017). Epidemiology of *salmonella*
*enterica* serotype Dublin infections among humans, United States, 1968–2013. Emerg. Infect. Dis..

[2] Kent E (2021). Control of Salmonella Dublin in a bovine dairy herd. J. Vet. Intern. Med..

[3] Holschbach CL, Peek SF (2018). Salmonella in dairy cattle. Vet. Clin. N. Am. Food Anim. Pract..

[4] McDonough PL, Fogelman D, Shin SJ, Brunner MA, Lein DH (1999). *Salmonella*
*enterica* serotype dublin infection: An emerging infectious disease for the Northeastern United States. J. Clin. Microbiol..

[5] Wang F (2022). Safety of the *Salmonella*
*enterica* serotype Dublin strain Sdu189-derived live attenuated vaccine—A pilot study. Front. Vet. Sci..

[6] Yin Y, Zhou D (2018). Organoid and enteroid modeling of Salmonella infection. Front. Cell. Infect. Microbiol..

[7] Vohra P, Vrettou C, Hope JC, Hopkins J, Stevens MP (2019). Nature and consequences of interactions between *Salmonella*
*enterica* serovar Dublin and host cells in cattle. Vet. Res..

[8] Haque A (2004). Early interactions of *Salmonella*
*enterica* serovar typhimurium with human small intestinal epithelial explants. Gut.

[9] Derricott H (2019). Developing a 3D intestinal epithelium model for livestock species. Cell Tissue Res..

[10] Shakya R, Jiménez-Meléndez A, Robertson LJ, Myrmel M (2023). Bovine enteroids as an in vitro model for infection with bovine coronavirus. Viruses.

[11] Blake R, Jensen K, Mabbott N, Hope J, Stevens J (2022). The development of 3D bovine intestinal organoid derived models to investigate *Mycobacterium*
*avium* ssp paratuberculosis pathogenesis. Front. Vet. Sci..

[12] Kar SK (2021). Organoids: A promising new in vitro platform in livestock and veterinary research. Vet. Res..

[13] Kawasaki M, Goyama T, Tachibana Y, Nagao I, Ambrosini YM (2022). Farm and companion animal organoid models in translational research: A powerful tool to bridge the gap between mice and humans. Front. Med. Technol..

[14] Holly MK (2020). *Salmonella*
*enterica* infection of murine and human enteroid-derived monolayers elicits differential activation of epithelium-intrinsic inflammasomes. Infect. Immun..

[15] Nickerson KP (2021). A versatile human intestinal organoid-derived epithelial monolayer model for the study of enteric pathogens. Microbiol. Spectr..

[16] In JG, Foulke-Abel J, Clarke E, Kovbasnjuk O (2019). Human colonoid monolayers to study interactions between pathogens, commensals, and host intestinal epithelium. J. Vis. Exp..

[17] Sutton KM, Orr B, Hope J, Jensen SR, Vervelde L (2022). Establishment of bovine 3D enteroid-derived 2D monolayers. Vet. Res..

[18] Töpfer E (2019). Bovine colon organoids: From 3D bioprinting to cryopreserved multi-well screening platforms. Toxicol. In Vitro.

[19] Kawasaki M, Dykstra GD, McConnel CS, Burbick CR, Ambrosini YM (2023). adult bovine-derived small and large intestinal organoids: In vitro development and maintenance. J. Tissue Eng. Regen Med..

[20] Marsh MN, Swift JA (1969). A study of the small intestinal mucosa using the scanning electron microscope. Gut.

[21] Miyazawa K (2010). Characterization of newly established bovine intestinal epithelial cell line. Histochem. Cell Biol..

[22] Sontheimer-Phelps A (2020). Human colon-on-a-chip enables continuous in vitro analysis of colon mucus layer accumulation and physiology. CMGH.

[23] Knodler LA (2010). Dissemination of invasive *Salmonella* via bacterial-induced extrusion of mucosal epithelia. Proc. Natl. Acad. Sci. U. S. A..

[24] Jepson MA, Collares-Buzato CB, Clark MA, Hirst BH, Simmons NL (1995). Rapid disruption of epithelial barrier function by *Salmonella*
*typhimurium* is associated with structural modification of intercellular junctions. Infect. Immun..

[25] Bolton AJ, Martin GD, Osborne MP, Wallis TS, Stephen J (1999). Invasiveness of *Salmonella*
*serotypes*
*typhimurium*, choleraesuis and dublin for rabbit terminal ileum in vitro. J. Med. Microbiol..

[26] Gagnon M, Zihler Berner A, Chervet N, Chassard C, Lacroix C (2013). Comparison of the Caco-2, HT-29 and the mucus-secreting HT29-MTX intestinal cell models to investigate Salmonella adhesion and invasion. J. Microbiol. Methods.

[27] Sun L (2020). Salmonella effector SpvB disrupts intestinal epithelial barrier integrity for bacterial translocation. Front. Cell. Infect. Microbiol..

[28] Eckmann L, Kagnoff MF (2001). Cytokines in host defense against *Salmonella*. Microbes Infect..

[29] Galán JE (2021). *Salmonella*
*typhimurium* and inflammation: A pathogen-centric affair. Nat. Rev. Microbiol..

[30] Hamilton CA (2018). Development of in vitro enteroids derived from bovine small intestinal crypts. Vet. Res..

[31] Felipe-López A, Hansmeier N, Danzer C, Hensel M (2023). Manipulation of microvillar proteins during *Salmonella*
*enterica* invasion results in brush border effacement and actin remodeling. Front. Cell. Infect. Microbiol..

[32] Forbester JL (2015). Interaction of *Salmonella*
*enterica* serovar typhimurium with intestinal organoids derived from human induced pluripotent stem cells. Infect. Immun..

[33] Zhang YG, Wu S, Xia Y, Sun J (2014). Salmonella-infected crypt-derived intestinal organoid culture system for host–bacterial interactions. Physiol. Rep..

[34] Otte JM, Podolsky DK (2004). Functional modulation of enterocytes by gram-positive and gram-negative microorganisms. Am. J. Physiol. Gastrointest. Liver Physiol..

[35] van der Hee B (2018). Optimized procedures for generating an enhanced, near physiological 2D culture system from porcine intestinal organoids. Stem Cell Res..

[36] Hoffmann P (2021). Intestinal organoid-based 2D monolayers mimic physiological and pathophysiological properties of the pig intestine. PLoS One.

[37] Kardia E (2021). Culture and differentiation of rabbit intestinal organoids and organoid-derived cell monolayers. Sci. Rep..

[38] Roodsant T (2020). A human 2D primary organoid-derived epithelial monolayer model to study host-pathogen interaction in the small intestine. Front. Cell. Infect. Microbiol..

[39] Ambrosini YM (2020). Recapitulation of the accessible interface of biopsy-derived canine intestinal organoids to study epithelial-luminal interactions. PLoS One.

[40] Srinivasan B (2015). TEER measurement techniques for in vitro barrier model systems. J. Lab. Autom..

[41] Ferruzza S, Rossi C, Sambuy Y, Scarino ML (2013). Serum-reduced and serum-free media for differentiation of caco-2 cells. ALTEX.

[42] Brown DR, Price LD (2007). Characterization of *Salmonella*
*enterica* serovar typhimurium DT104 invasion in an epithelial cell line (IPEC J2) from porcine small intestine. Vet. Microbiol..

[43] VanDussen KL (2015). Development of an enhanced human gastrointestinal epithelial culture system to facilitate patient-based assays. Gut.

[44] Fernando EH (2017). A simple, cost-effective method for generating murine colonic 3D enteroids and 2D monolayers for studies of primary epithelial cell function. Am. J. Physiol. Gastrointest. Liver Physiol..

[45] Barrila J (2017). Three-dimensional organotypic co-culture model of intestinal epithelial cells and macrophages to study *Salmonella*
*enterica* colonization patterns. NPJ Microgravity.

[46] Kawasaki M, Ambrosini YM (2024). Accessible luminal interface of bovine rectal organoids generated from cryopreserved biopsy tissues. PLoS One.

[47] Lacroix-Lamandé S (2023). Differential *Salmonella*
*typhimurium* intracellular replication and host cell responses in caecal and ileal organoids derived from chicken. Vet. Res..

[48] Uzzau S (2001). Salmonella enterica serovar-host specificity does not correlate with the magnitude of intestinal invasion in sheep. Infect. Immun..

[49] Eckmann L, Kagnoff MF, Fierer J (1993). Epithelial cells secrete the chemokine interleukin-8 in response to bacterial entry. Infect. Immun..

[50] Santos RL, Zhang S, Tsolis RM, Bäumler AJ, Adams LG (2002). Morphologic and molecular characterization of *Salmonella*
*typhimurium* infection in neonatal calves. Vet. Pathol..

[51] Nunes JS (2010). Morphologic and cytokine profile characterization of *Salmonella*
*enterica* serovar typhimurium infection in calves with bovine leukocyte adhesion deficiency. Vet. Pathol..

[52] Casaux M (2023). Epidemiological and clinicopathological findings in 15 fatal outbreaks of salmonellosis in dairy calves and virulence genes in the causative *Salmonella*
*enterica* Typhimurium and Dublin strains. Braz. J. Microbiol..

[53] Klimpel GR, Asuncion M, Haithcoat J, Niesel DW (1995). Cholera toxin and *Salmonella*
*typhimurium* induce different cytokine profiles in the gastrointestinal Tract. Infect. Immun..

[54] Sato T (2011). Long-term expansion of epithelial organoids from human colon, adenoma, adenocarcinoma, and Barrett’s epithelium. Gastroenterology.

[55] Strengert M, Knaus UG (2011). 13 Analysis of epithelial barrier integrity in polarized lung epithelial cells. Methods Mol. Biol..

[56] Khare S (2009). Early phase morphological lesions and transcriptional responses of bovine ileum infected with *Mycobacterium*
*avium* subsp. paratuberculosis. Vet. Pathol..

[57] Zhan K, Yang TY, Chen Y, Jiang MC, Zhao GQ (2020). Propionate enhances the expression of key genes involved in the gluconeogenic pathway in bovine intestinal epithelial cells. J. Dairy Sci..

[58] Koch F (2019). Heat stress directly impairs gut integrity and recruits distinct immune cell populations into the bovine intestine. Proc. Natl. Acad. Sci. U. S. A..

[59] Charavaryamath C (2011). Mucosal changes in a long-term bovine ileal segment model following removal of ingesta and microflora. Gut Microbes.

[60] Ontsouka EC, Korczak B, Mammon HM, Blum JW (2004). Real-time PCR quantification of bovine lactase mRNA: Localization in the gastrointestinal tract of milk-fed calves. J. Dairy Sci..

[61] Inderwies T, Pfaffl MW, Meyer HHD, Blum JW, Bruckmaier RM (2003). Detection and quantification of mRNA expression of α- and β-adrenergic receptor subtypes in the mammary gland of dairy cows. Domest. Anim. Endocrinol..

[62] Connelly MK, Hernandez LL (2021). Elevated serotonin alters whole-blood expression of serotonin receptor and metabolism genes in the lactating dairy cow. JDS Commun..

[63] Rinaldi M, Ceciliani F, Lecchi C, Moroni P, Bannerman DD (2008). Differential effects of α1-acid glycoprotein on bovine neutrophil respiratory burst activity and IL-8 production. Vet. Immunol. Immunopathol..

