# Supplementary material for: Pathogen-epithelium interactions and inflammatory responses in Salmonella Dublin infections using ileal monolayer models derived from adult bovine organoids
Source: Sci Rep. 2024 May 20;14:11479. doi: 10.1038/s41598-024-62407-2 (PMC11106274; doi:10.1038/s41598-024-62407-2)
Supplement: Supplementary file 1 — Supplementary Information. [file 41598_2024_62407_MOESM1_ESM.pdf]

Pathogen-Epithelium Interactions and Inflammatory Responses in *Salmonella* Dublin Infections using Ileal Monolayer Models Derived from Adult Bovine Organoids

Minae Kawasaki[1], Craig S. McConnel[1], Claire R. Burbick[2], and Yoko M. Ambrosini[1*]

[1] Department of Veterinary Clinical Sciences, College of Veterinary Medicine, Washington State University, Pullman, Washington, United States of America
[2] Department of Veterinary Microbiology and Pathology, College of Veterinary Medicine, Washington State University, Pullman, Washington, United States of America

*Corresponding author:
E-mail: yoko.ambrosini@wsu.edu (YMA)

Supplementary information

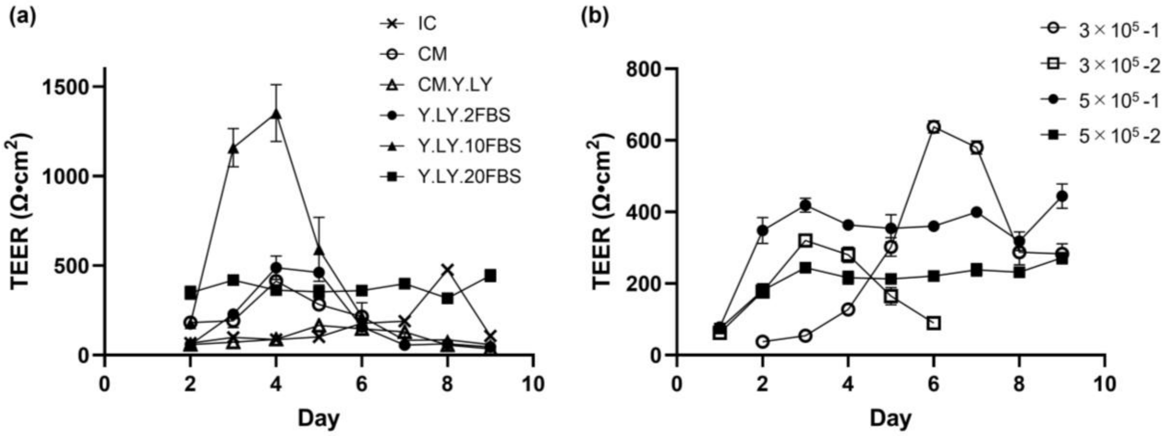

Figure S1. Optimization of bovine ileal organoid-derived monolayer culture conditions. (a) Two organoid culture media (IC and CM) with or without supplementations (Y, LY and FBS) were tested for their effectiveness to generate stable bovine ileal organoid-derived monolayers. Stability of the monolayers was assessed based on the TEER. Cells were seeded at 5×10^5 cells/well in all conditions. IC: Intesticult, CM: DMEM/F12-based organoid culture medium, Y: Y-27632, LY: LY2157299, FBS: 2, 10 or 20% fetal bovine serum. (b) Low (3×10^5 cells/well) and high (5×10^5 cells/well) cell seeding densities were compared. Cells were cultured in the optimized monolayer culture medium, which is DMEM/F12-based organoid culture medium supplemented with Y, LY, and 20% FBS. Results are expressed as mean ± s.e.m. obtained from an independent experiment with at least two technical replicates using three biological replicates.

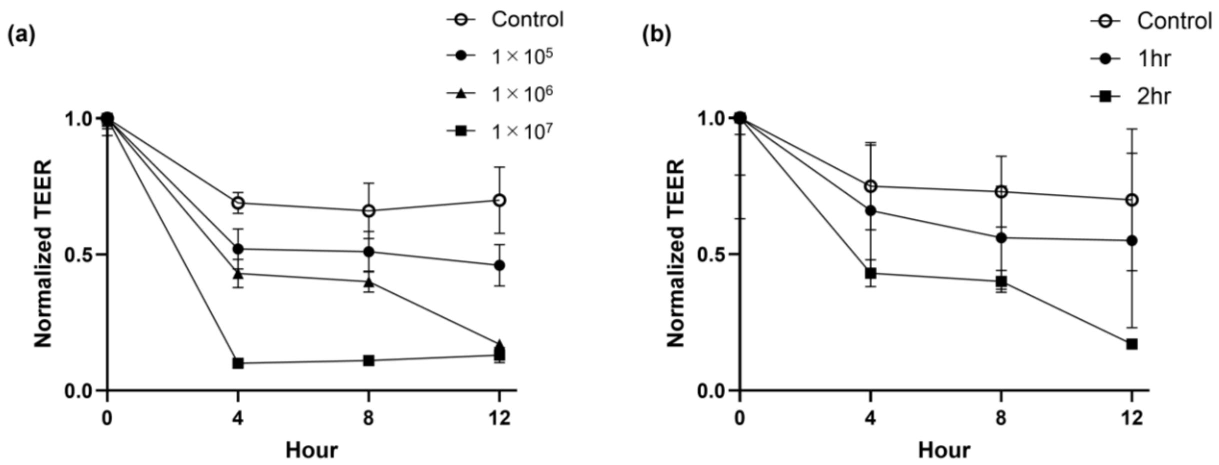

Figure S2. Optimization of *S.* Dublin infection conditions in bovine ileal organoid-derived monolayers. (a) Low (1 × 10^5 CFU/well), medium (1 × 10^6 CFU/well) and high (1 × 10^7 CFU/well) concentrations of *S.* Dublin were apically exposed to bovine ileal organoid-derived monolayers. Bacteria were incubated with the monolayers in optimized monolayer culture medium without antibiotics for 2 hours before treatment with 50 μg/mL gentamicin. (b) The medium concentration of *S.* Dublin was incubated in the antibiotic-free culture medium for either one or two hours before they were treated with 50 μg/mL gentamicin. Impact of *S.* Dublin infection on the monolayers was assessed by measuring the TEER over 12 hours. The TEER values at each time point were normalized by the pre-infection value. Results are expressed as mean ± s.e.m. obtained from an independent experiment with at least two technical replicates using three biological replicates.

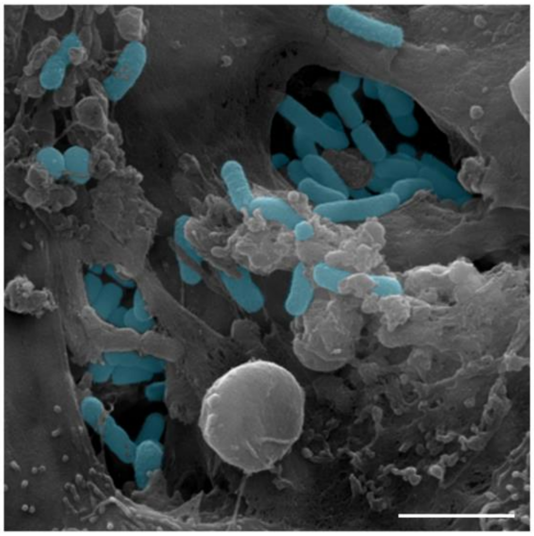

Figure S3. Intracellular invasion and replication of *S*. Dublin in bovine ileal organoid-derived monolayers. A zoomed-in image of Figure 3b was pseudo-colored to enhance visualization of *Salmonella* invasion and replication within the bovine ileal monolayer. Bar: 2 µm.

Table S1. Bacterial growth in apical and basolateral culture media.

Bacterial growth	1-hr	2-hr	24-hr
Apical medium	+	-	+
Basolateral medium	-	-	-

Culture media in the apical and basolateral compartments were inoculated on LB agar and cultured overnight at 37°C at 1-, 2- and 24-hour post infection. Bacterial growth was noted in the apical media collected at 1- and 24-hour post infection, whereas no growth was observed in the medium collected at 2-hour, indicating that bacteria detected at 24-hour originated from those which invaded the epithelial cells during the first hour of infection. No bacterial growth was detected in basolateral culture media collected at all time points.

Table S2. Signalment of tissue donors and identification of donors used for each analysis.

Animal ID	Breed	Sex	Estimated Age (Months)	PC	IF	SEM	TEM	TEER	P_{app}	RT-qPCR	ELISA	Bac Enum.
1	Hereford	Steer	18	×	×		×	×	×	×	×	×
2	Angus × Charolais	Steer	18		×			×	×	×	×	×
3	Charolais	Steer	18	×	×	×		×				
4	Angus	Heifer	15-18			×	×	×	×	×	×	×

"×" indicates the donors used to generate the data presented in the main and supplementary figures. Cells from passages 5 through 13 were utilized in this study. PC: phase contrast microscopy, IF: immunofluorescence staining, SEM: scanning electron microscopy, TEM: transmission electron microscopy, TEER: transepithelial electrical resistance measurement, P_{app}: paracellular permeability assay, RT-qPCR: reverse transcription quantitative polymerase chain reaction, ELISA: enzyme linked immunosorbent assay, Bac Enum: bacterial enumeration.

Table S3. Organoid and monolayer culture media used in this study.

Reagent	Final Concentration	Organoid Culture Medium	Monolayer Culture Medium
Advanced DMEM/F12	NA	+	+
Noggin Conditioned Medium	10% (v/v)	+	+
R-Spondin Conditioned Medium	20% (v/v)	+	+
Recombinant Murine Wnt-3a	100 ng/mL	+	+
A-83-01	500 nM	+	+
B27	1 ×	+	+
Murine EGF	50 ng/mL	+	+
Gastrin	10 nM	+	+
N2	1 ×	+	+
Nicotinamide	10 mM	+	+
N-Acetyl-L-cysteine	1 mM	+	+
SB202190	10 μM	+	+
Primocin	100 μg/mL	+	-
Penicillin/Streptomycin	1 ×	+	-
GlutaMAX	2 mM	+	+
HEPES	10 mM	+	+
CHIR99021	100 nM	+*	-
Y-27632	10 μM	+*	+
LY2157299	500 nM	-	+
Fetal Bovine Serum	20% (v/v)	-	+

"*" indicates removal of the reagent from the culture medium after the first few days of culture.

Table S4. Primers for evaluation of gene expression levels of bovine ileal organoid-derived monolayers.

Gene	Gene Name	Forward	Reverse	Reference
ACTB	*β-actin*	CTAGGCACCAGGGCGTAATG	CCACACGGAGCTCGTTGTAG	[1]
CHGA	*Chromogranin A*	GGGACACTGAGGTGATGAAG	GTCGCAGGATTGAGAGGAT	[2]
FABP2	*Fatty acid binding protein 2*	GGAGGGAGATAAACTTGTCGG	ATCTGTGTTCTGGGCAATGC	[3]
GAPDH	*Glyceraldehyde-3-phosphate dehydrogenase*	ATCTCGCTCCTGGAAGATG	TCGGAGTGAACGGATTCG	[4]
IL6	*Interleukin 6*	ACCCCAGGCAGACTACTTCT	GCAAATCGCCTGATTGAACCC	[5]
IL8	*Interleukin 8*	TGCTTTTTTGTTTTCGGTTTTTG	AACAGGCACTCGGGAATCCT	[6]
LGR5	*Leucine rich repeat containing G protein-coupled receptor 5*	ACTTTCCAGCAGTTGTTCAGC	GAATAGACGACAGGCGGTTG	[2]
LYZC	*Lysozyme C*	TTCCTTTCTGTTGCTGTCCA	AGCCATCCAGTCCAAGTTTC	[2]
MUC2	*Mucin 2*	TTCGACGGGAGGAAGTACAC	TTCACCGTCTGCTCATTCAG	[4]
RPL0	*Ribosomal protein L0*	CAACCCTGAAGTGCTTGACAT	AGGCAGATGGATCAGCCA	[5]
TNFA	*Tumor necrosis factor alpha*	AGAGGGAAGAGCAGTCCCCAG	TTCACACCGTTGGCCATGAG	[5]

References

1. Charavaryamath, C. *et al.* Mucosal changes in a long-term bovine ileal segment model following removal of ingesta and microflora. *Gut Microbes* **2**, 134–144 (2011).
2. Sutton, K. M., Orr, B., Hope, J., Jensen, S. R. & Vervelde, L. Establishment of bovine 3D enteroid-derived 2D monolayers. *Vet Res* **53**, 15 (2022).
3. Zhan, K., Yang, T. Y., Chen, Y., Jiang, M. C. & Zhao, G. Q. Propionate enhances the expression of key genes involved in the gluconeogenic pathway in bovine intestinal epithelial cells. *J Dairy Sci* **103**, 5514–5524 (2020).
4. Shakya, R., Jiménez-Meléndez, A., Robertson, L. J. & Myrmel, M. Bovine Enteroids as an In Vitro Model for Infection with Bovine Coronavirus. *Viruses* **15**, 635 (2023).
5. Koch, F. *et al.* Heat stress directly impairs gut integrity and recruits distinct immune cell populations into the bovine intestine. *Proc Natl Acad Sci U S A* **116**, 10333–10338 (2019).
6. Khare, S. *et al.* Early phase morphological lesions and transcriptional responses of bovine ileum infected with Mycobacterium avium subsp. paratuberculosis. *Vet Pathol* **46**, 717–728 (2009).
